# Latent harmony in dicarbon between VB and MO theories through orthogonal hybridization of 3σ_g_ and 2σ_u_

**DOI:** 10.1039/c5sc03437j

**Published:** 2015-10-27

**Authors:** Ronglin Zhong, Min Zhang, Hongliang Xu, Zhongmin Su

**Affiliations:** a Institute of Functional Material Chemistry & Local United Engineering Lab for Power Battery , Faculty of Chemistry , Northeast Normal University , Changchun 130024 , China . Email: mzhang@nenu.edu.cn ; Email: zmsu@nenu.edu.cn

## Abstract

Besides the classic double bond scheme, several novel schemes have been proposed to describe the nature of the chemical bond in dicarbon (C_2_), including a quadruple bond and a singlet diradical state. The results from a symmetry-broken CASSCF(8,8)/aug-cc-pVTZ study present a harmony between MO and VB theories, based on the orthogonal hybridization of the 3σ_g_ and 2σ_u_ orbitals together with the other six pristine valence orbitals. This scheme achieves the same bonding energy, *R*_C–C_, *ω*_e_ and one electron density as that from the eight pristine valence orbitals. A quadruple bond scheme, identical to Prof. Shaik's result from VB theory, is achieved with the 4^th^ bond energy in the range of 12.8–27.6 kcal mol^–1^. Meanwhile, the weight of a singlet open-shell configuration is the highest among all the possible configurations.

## Abstract 2

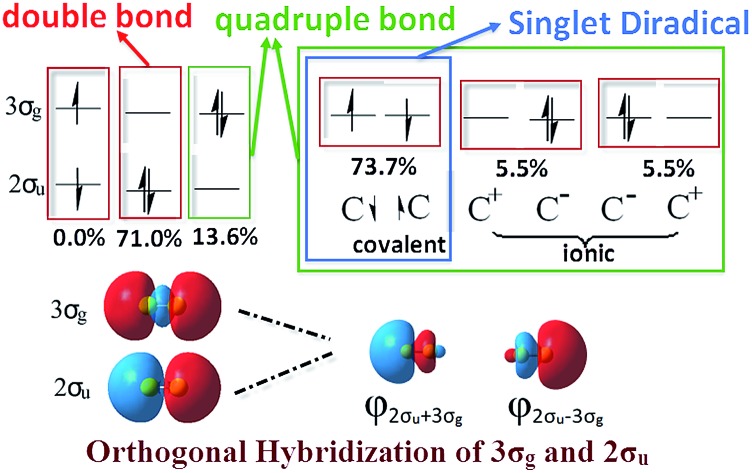
Besides the classic double bond scheme, several novel schemes have been proposed to describe the nature of the chemical bond in dicarbon, including a quadruple bond and a singlet diradical state. The results presented here show a harmony between MO and VB theories.

## Introduction

Dicarbon (C_2_) is a simple molecule with just two atoms. However, it has aroused many fundamental questions, fascinating mysteries and active discussions in chemistry.^1^–^6^ It is a colourless gas and is very unstable. A tiny quantity of C_2_ can be prepared from electric arc strikes, and a good amount of C_2_ can exist in comets, stellar atmospheres, blue hydrocarbon flames, *etc.* The C–C bond length (*R*_C–C_) is 1.243 Å in its ^1^Σ+g ground state, and the corresponding vibration frequency (*ω*_e_) is 1855 cm^–1^.^2^,^7^ At least another 12 excited states have been observed experimentally and the *R*_C–C_ values are found to be in a large range of 1.23 Å to 1.53 Å.^2^,^4^ Among them, two *R*_C–C_ values bear shorter bond lengths in the excited states (^3^Σ+u and ^1^Σ+u) than in the ground state. Similar shorter bond lengths in the excited states have also been reported in its cation and anion (C_2_^+^ and C_2_^–^).^8^–^11^ The *R*_C–C_ of the dicarbide ion (C_2_^2–^) in crystalline calcium carbide and lithium carbide is shorter than 1.20 Å,^12^,^13^ which is generally accepted as a traditional triple bond (1σ + 2π bonds) analogue of N_2_.

**Fig. 1:**
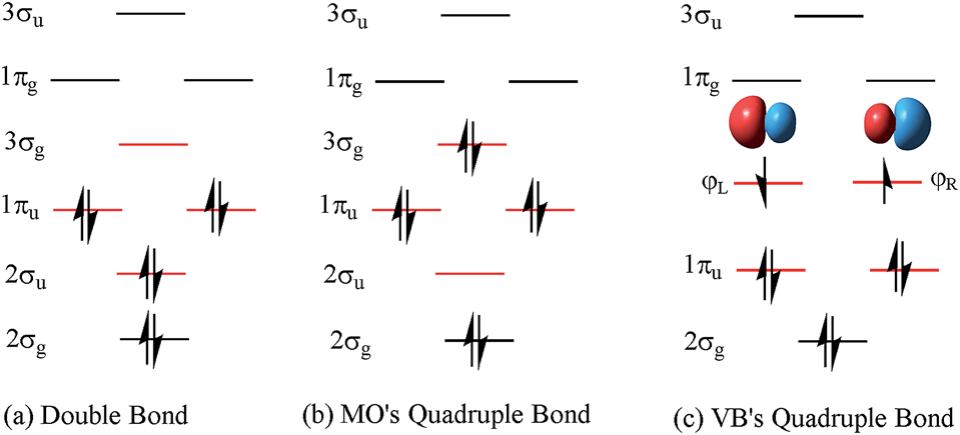
The configurations of MO’s double bond, MO’s quadruple bond and VB's quadruple bond schemes in C_2_. The difference in the order of the occupied orbitals is highlighted with red lines.

It is worthy of note that the ground *R*_C–C_ distance of C_2_ is 1.243 Å, shorter than the length of any classic C=C double bond (1σ + 1π bonds), such as in ethylene.^14^ Hence, Prof. Shaik^15^ pointed out that suspended π bonds may be responsible, since they prefer shorter lengths than σ bonds. The essential point of this assumption is that the occupied number of the 2σ_u_ antibond is approximately equal to that of the 2σ_g_ bond. However, 2σ_u_ is a weak antibonding orbital due to a lower occupied number, which can not counteract the stronger bonding of 2σ_g_. Furthermore, the *ω*_e_ value of C_2_ is higher than that of ethylene. Hence, soon after, the nature of the C_2_ bond was said to approach that of a triple bond (C≡C).^16^,^17^ Based on the characteristics of a triple bond in C_2_, a scheme of a triple bond plus weak coupling by a pair of opposite spinning electrons was proposed in valence bond (VB) theory.^18^ The opposite spinning electron coupling energy was found to be ∼12–20.2 kcal mol^–1^ (ref. 19 and 20) at various levels of the theory. In this context, the corresponding 4^th^ bonding scheme of C_2_ (Fig. 1c) was proposed with VB theory.^19^

A quadruple bond in C_2_ is certainly reasonable based on quantum mechanics,^19^,^21^ similar to that in [1.1.1]-propellane.^22^ However, whether it is the best picture to describe the ground state of C_2_ or not has aroused many active discussions.^21^,^23^–^26^ According to traditional molecular orbital (MO) theory, the quadruple bond configuration of C_2_ can be achieved by doubly exciting 2σ_u_ electrons to the 3σ_g_ orbitals. However, a calculation at the CASSCF(8,8)/cc-pVTZ level indicates that the weight of the double bond state (Fig. 1a) is 71.0% while the weight of the 2σ_u_ → 3σ_g_ quadruple bond counterpart (Fig. 1b) is only 13.6%.^21^ On the other hand, the weak 4^th^ bond (the inset in Fig. 1c) was proposed by inequivalent *hybridization* of the 3σ_g_ and 2σ_u_ occupied states, covering only MO double and quadruple bond configurations, which does not seem perfect. Even in an egalitarian mode, the corresponding bond order of C_2_ is between two and three. What is the dominant configuration like if orthogonal hybridization of 3σ_g_ and 2σ_u_ is utilized and 100% weight of multi-configurational self-consistent field (MCSCF) is covered in the calculation? Is there any other configuration that can possess a higher weight in the ground state (^1^Σ+g) of C_2_ among all the possible configurations?

## Results and discussion

As we know, the ground state of C_2_ is generally accepted as double π bonds in MO theory (shown in Fig. 1a). However, the quasi-degeneracy of the 2σ_u_, 3σ_g_ and 1π_u_ orbitals is well known in C_2_ and its ions, which results in many low-lying excited states of C_2_, C_2_^–^ and C_2_^+^.^1^,^2^,^4^ The antiferromagnetic diradical characteristic of C_2_ has also been proposed by the finite-difference pseudopotential method, local spin analysis and VB theory.^24^–^26^ Silicon resides in the same column of the periodic table as carbon. The singlet diradical characteristic on a silicon (100) surface is well known.^27^,^28^ Moreover, dicarbon is a very unstable molecule with a short lifetime, which is easy to dimerize into C_4_ for instance.

**Fig. 2:**
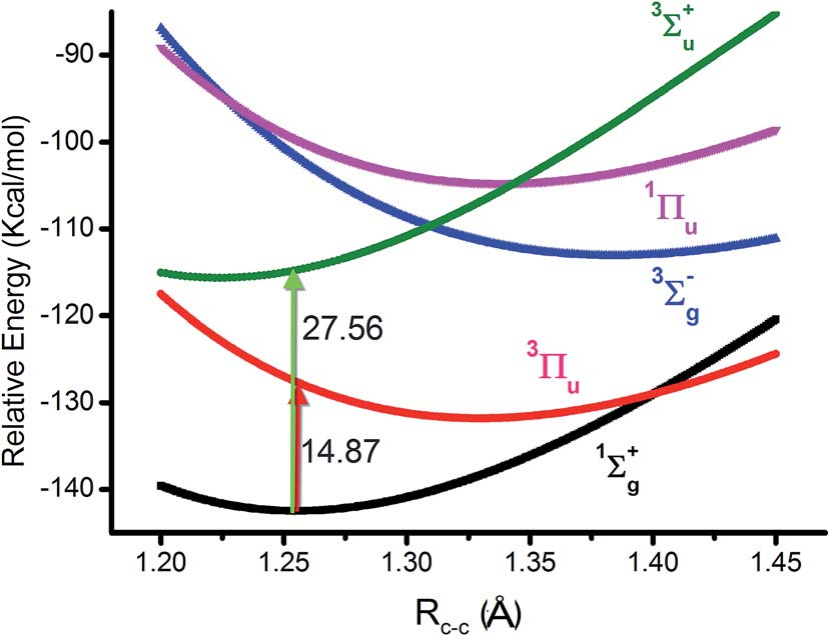
Plots of C_2_ potential energy curves of ^1^Σ+g state and four (^3^Π_u_, ^3^Σ+u, ^3^Σ–g and ^1^Π_u_) excited states at CASSCF(8,8)/aug-cc-pVTZ level. The bonding energy is relative to two isolated ^3^P carbon atoms at CASSCF(4,4)/aug-cc-pVTZ level. The vertical transition energies from ^1^Σ+g state are also shown.

As mentioned above, CASSCF(8,8)/aug-cc-pVTZ was used in our study because C_2_ has multi-reference configurations in nature. In the beginning, the level was benchmarked for the ^1^Σ+g ground state (KK2σ2g1π2xu1π2yu2σ2u) and four low-lying (^3^Π_u_, ^3^Σ+u, ^3^Σ–g and ^1^Π_u_) excited states. All the potential energy curves are plotted in Fig. 2. Furthermore, the important data of these optimized states contrasting to the experimental data are listed in Table 1.

**Table 1:** The most important theoretical data of the five states, including *R*_C–C_s (Å) and vibrational frequencies (*ω*_e_, cm^–1^), together with their experimental data (shown in bracket)

	^1^Σ+g	^3^Π_u_	^3^Σ+u	^3^Σ–g	^1^Π_u_
***R*_C–C_ (Å)**	1.255 (1.243)	1.330 (1.312)	1.224 (1.230)	1.384 (1.369)	1.338 (1.318)
**Δ*E*_relat._ (kcal mol^–1^)**	–142.5 (–142)	–131.8	–114.9	–113.1	–104.7
***ω*_e_ (cm^–1^)**	1839 (1855)	1599 (1641)	1975 (2084)	1436 (1470)	1553 (1608)
**Dominant configuration**	KK2σ2g	KK2σ2g2σ2u	KK2σ2g1π2xu	KK2σ2g2σ2u	KK2σ2g2σ2u
	1π2xu1π2yu2σ2u	1π2xu1παyu3σαg	1π2yu2σαu3σβg	3σ2g1παxu1παyu	1π2xu1παyu3σβg
**EBO**	2.15	1.90	2.74	1.92	1.90
**Weight**	71.0%	87.5%	85.1%	93.0%	89.9%
**One electron density**	0.014*e* (3σ_u_)	0.020*e* (3σ_u_)	0.016*e* (3σ_u_)	0.026*e* (3σ_u_)	0.027*e* (3σ_u_)
	0.115*e* (1π_yg_)	0.074*e* (1π_yg_)	0.109*e* (1π_yg_)	0.048*e* (1π_yg_)	0.041*e* (1π_yg_)
	0.115*e* (1π_xg_)	0.098*e* (1π_xg_)	0.109*e* (1π_xg_)	0.048*e* (1π_xg_)	0.097*e* (1π_xg_)
	0.393*e* (3σ_g_)	0.965*e* (1π_yu_)	0.976*e* (3σ_g_)	0.990*e* (1π_xu_)	0.985*e* (1π_yu_)
	1.602*e* (2σ_u_)	1.045*e* (3σ_g_)	1.031*e* (2σ_u_)	0.990*e* (1π_yu_)	1.023*e* (3σ_g_)
	1.888*e* (1π_yu_)	1.905*e* (1π_xu_)	1.891*e* (1π_yu_)	1.956*e* (2σ_u_)	1.912*e* (1π_xu_)
	1.888*e* (1π_xu_)	1.910*e* (2σ_u_)	1.891*e* (1π_xu_)	1.966*e* (3σ_g_)	1.933*e* (2σ_u_)
	1.984*e* (2σ_g_)	1.982*e* (2σ_g_)	1.977*e* (2σ_g_)	1.978*e* (2σ_g_)	1.982*e* (2σ_g_)

The calculated *R*_C–C_ and *ω*_e_ values of the ^1^Σ+g state are close to the experimental values. For example, the bonding energy of the ^1^Σ+g state is –142.5 kcal mol^–1^, which is almost equivalent to the ideal *R*_C–C_ bonding breakage of C_2_ based on the heats of formation.^23^ The *R*_C–C_ differences of the other four states compared to the experimental data are only less than 0.02 Å. These results clearly show that the results from the CASSCF(8,8)/aug-cc-pVTZ level are reliable. In the ground state, 3σ_g_ is a weak bond with a one electron density of ∼0.4*e*. It contributes somewhat to the stabilization of C_2_. Hence, a triple bond scheme of C_2_ is also reasonable.^16^,^17^ However, the one electron density of the 2σ_u_ orbital is ∼1.6*e*, which is ∼1.2*e* higher than that of the 3σ_g_ orbital. If the populations of all the other three valence orbitals (1π_xg_, 1π_yg_ and 3σ_u_) are included, the relative 2σ_u_ antibond electrons are still ∼1.0*e* higher. In this context, the quadruple bond scheme is hard to be accepted by naive application of MO theory.

**Fig. 3:**
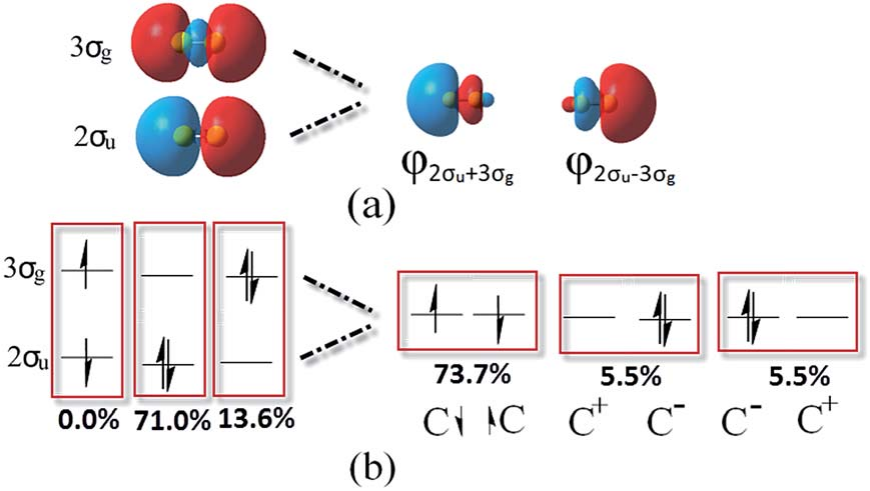
Equivalent *hybridized* orbitals of φ_L_ and φ_R_ from 2σ_u_ and 3σ_g_ localized around a single C atom (a) and the corresponding potential occupied styles before and after hybridization (b).

Based on the traditional valence MOs, it is hard to interpret the singlet diradical characteristic of the C_2_ ground state, while it has been shown through the finite-difference pseudopotential method and LSA analysis.^25^,^26^ Even though a singlet diradical state (KK2σ2g1π4u2σ↓u3σ↑g) was achieved, it is still a ^1^Σ+u state. The diagram of 2σ↓u3σ↑g occupation is shown in the left part of Fig. 3. As a result, the MO and VB theories fall into an apparent contradiction. In our opinion, this just indicates that opposite spinning electrons do not locate around the C–C bonds, which is in accordance with Prof. Shaik's proposal. However, the difficulty with the VB interpretation of the full configuration interaction (CI) wave function may be due to the nonorthogonal transform and the neglect of ∼15% weight of the configuration state functions (CSFs). Can VB quadruple bond schemes be reliable through the reformed valence MO orbitals? Is it feasible to describe the singlet diradical characteristic of C_2_ through the reformed valence MO orbitals simultaneously?

We hereby propose another scheme through hybrid orbitals of the 2σ_u_ and 3σ_g_ orbitals to φ_2σ_u_+3σ_g__(φ_L_) and φ_2σ_u_–3σ_g__(φ_R_), which was used as the initial active orbital for the CASSCF calculation. It is worthy of note that the φ_L_ and φ_R_ orbitals ensure that the single-occupied electrons are located outside of the C–C bond (right part of Fig. 3a). Besides, the other six pristine valence orbitals were maintained. Subsequently, the potential energy curve of the ground state of C_2_ was re-scanned at the same level with our orbitals. Compared with Prof. Shaik's strategy, the eight CAS orbitals were all orthogonal and the SCFs covered 100% of the weight. As expected, the new results show that the bonding energy, *R*_C–C_, *ω*_e_ and one electron density are all the same as the values from the eight pristine valence orbitals (in the first column of Table 1). This is reasonable because the final result is only determined by the one electron densities in the original natural orbitals and the corresponding gradient analysis when all the CSFs are included in the simulations. CASSCF(8,8) and VBSCF(1764) span the same space of 1764 configurations in MO and VB theories, separately. Theoretically, the same electron correlation energy and bonding energy can be achieved if the same orbitals are adopted in the simulation. Unfortunately, orthogonal orbitals are adopted in the former, but the nonorthogonal orbitals are adopted in the latter.^18^

**Fig. 4:**
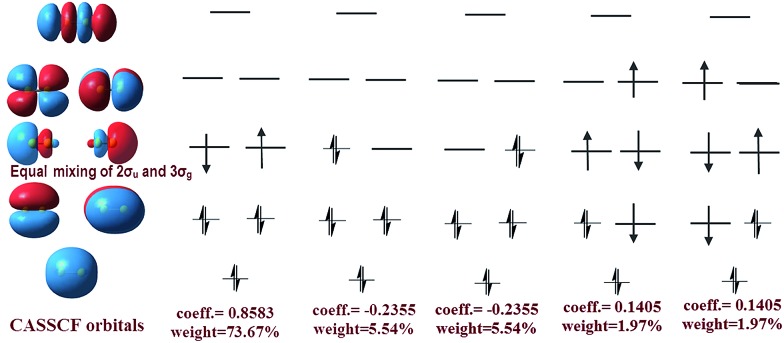
Left: CASSCF orbitals, including φ_L_ and φ_R_. Right: The corresponding five most important configurations of a full-valence CASSCF(8,8)/aug-cc-PVTZ calculation of C_2_, showing the coefficients and the weights of the configurations.

The difference between our and Prof. Frenking's CASSCF schemes^21^ is the input active orbitals and their corresponding CSF weights, which are affected by the input orbital styles. The five highest weight configurations of our scheme are shown in Fig. 4. The weight of the highest configuration (KK2σ2g1π2xu1π2yuφ↓Lφ↑R) is 73.7%, around 2.7% higher than that of Prof. Frenking's (KK2σ2g1π2xu1π2yu2σ2u). The results elucidate that the spin-localization of the 2σ_u_ electrons is, indeed, outside of the C–C bond, in accordance with the VB view.^18^,^19^ If the bonding nature is estimated by the highest weight configuration, the ground state of C_2_ is inclined to be a singlet diradical due to a little higher weight than the traditional double bond configuration.

However, with our understanding from VB theory, the first three configurations have to be added up, since there is a combination of a major covalent structure (C^↓^–C^↑^), and two minor ionic ones (C^–^–C^+^ and C^+^–C^–^). They all belong to the 4^th^ bond. To our surprise, the total weight of the three highest configurations is equal to the weight sum of the classic MO double and quadruple bond configurations (Fig. 3b), namely the initial value in Prof. Shaik's nonorthogonal scheme. Hence, the corresponding covalent component of the 4^th^ bond is 84.8% (close to the weight of the ^3^Π_u_ and ^3^Σ–u states). The opposite spin coupling energy between φ↓Lφ↑R can not be achieved directly in our orthogonal schemes. If the vertical excited energy from the ^1^Σ+g to ^3^Π_u_ states (*R*_C–C_ is 1.253 Å) is the minimum to break the coupling energy of φ_L_ and φ_R_, the relevant value is 12.8–15.1 kcal mol^–1^ (depending on the calculated weight), which is also consistent with the VB results. If the correct decoupled triplet state ^3^Σ+u is utilized as the electron spin-flip energy, the maximum of the 4^th^ bonding energy is 27.56 kcal mol^–1^. Hence, the VB 4^th^ bond based on Lewis electron pairing is reasonable. The results of our scheme implement an inherent harmony between VB and MO theories.

Furthermore, we focus on the Effective Bond Order (EBO) based on the one electron density of the ground state in Table 1.^29^ The EBO of 2σ_g_–2σ_u_ is 0.191 and that of 3σ_g_–3σ_u_ is 0.190. How to define their bond orders is still a problem. No σ bond or two weak σ bonds? If no σ bond, then it is a classic double bond scheme. If two weak σ bonds, then it is another quadruple bond scheme. Anyway, the EBO results at least demonstrate that the two π bonds contribute most to the bonding energy if the ^3^P state of the carbon atom is a starting point. That is the reason why the C–C bonding breakage of C_2_ is smaller than that of ethylene (–172 kcal mol^–1^). In our simulation, the highest EBO is 2.74 for the ^3^Σ+u state, because of the occupied number of 0.976*e* in the 3σ_g_ orbitals. Some distribution of 3σ_g_ locates between the two carbon atoms. A similar contribution is from the lower occupied number of 1.031*e* in the 2σ_u_ orbitals. Hence, its *R*_C–C_ value is ∼0.03 Å shorter than the *R*_C–C_ value of the ground state, and the corresponding *ω*_e_ value is the highest.

In the end, we would like to say a little more about the nature of the chemical bond in C_2_ with an ancient Chinese poem about Mountain Lu written by Su Shi: “*It's a range viewed in face and peaks from the side. Assuming different shapes viewed from far and wide. Of the Mountain Lu we cannot make out the true face. For we are lost in the heart of the very place*”.^30^ We are shown that the shape and scenery of Mountain Lu are different from different perspectives. Similarly, the understanding of “*the most rigorous theory*”^23^ for C_2_ perhaps depends on the various viewpoints of chemists.

## Conclusions

In summary, a quadruple bond scheme identical to Prof. Shaik's result from VB theory is achieved, which is related to its *R*_C–C_ length. Meanwhile, the weak 4^th^ bond or the singlet diradical characteristic of C_2_ is also easy to be understood, and is related to its instable/reactive nature. Our study conquers the shortcoming of traditional valence MOs. It is worthy of note that C_2_ must have multi-reference configurations in nature due to having no energy difference among these schemes. The only difference is the dominant contribution in the total CSFs, and how to understand them.

## Methodology

Based on the calculations in previous references,^21^,^23^ the precision achieved by CASSCF(8,8), which covers the CSFs excited including all the valence electron orbitals, is as good as full CI, since it is commonly recognized that the weights of the inner 1σ2g1σ2u orbitals are always 100% in full CI simulations. The configurations of the C_2_ electronic states are constructed directly from combinations of natural atomic orbitals in our simulations, because natural orbitals, as a particularly efficient choice, possess the unique advantage of minimizing the mixing effect of the 2s–2p orbitals in carbon and eliminating the diversification of LCAO–MOs in MCSCF simulations.^31^–^34^ All the calculations were performed mainly based on the GAUSSIAN 09 program package.^35^
